# A meta-analysis of the abscopal effect in preclinical models: Is the biologically effective dose a relevant physical trigger?

**DOI:** 10.1371/journal.pone.0171559

**Published:** 2017-02-21

**Authors:** Raffaella Marconi, Silvia Strolin, Gianluca Bossi, Lidia Strigari

**Affiliations:** 1 Laboratory of Medical Physics and Expert Systems, Regina Elena National Cancer Institute, Rome, Italy; 2 Translational Research Area, Regina Elena National Cancer Institute, Rome, Italy; Northwestern University Feinberg School of Medicine, UNITED STATES

## Abstract

**Background:**

Preclinical in vivo studies using small animals are considered crucial in translational cancer research and clinical implementation of novel treatments. This is of paramount relevance in radiobiology, especially for any technological developments permitted to deliver high doses in single or oligo-fractionated regimens, such as stereotactic ablative radiotherapy (SABR). In this context, clinical success in cancer treatment needs to be guaranteed, sparing normal tissue and preventing the potential spread of disease or local recurrence. In this work we introduce a new dose-response relationship based on relevant publications concerning preclinical models with regard to delivered dose, fractionation schedule and occurrence of biological effects on non-irradiated tissue, abscopal effects.

**Methods:**

We reviewed relevant publications on murine models and the abscopal effect in radiation cancer research following PRISMA methodology. In particular, through a log-likelihood method, we evaluated whether the occurrence of abscopal effects may be related to the biologically effective dose (BED). To this aim, studies accomplished with different tumor histotypes were considered in our analysis including breast, colon, lung, fibrosarcoma, pancreas, melanoma and head and neck cancer. For all the tumors, the *α* / *β* ratio was assumed to be 10 Gy, as generally adopted for neoplastic cells.

**Results:**

Our results support the hypothesis that the occurrence rate of abscopal effects in preclinical models increases with BED. In particular, the probability of revealing abscopal effects is 50% when a BED of 60 Gy is generated.

**Conclusion:**

Our study provides evidence that SABR treatments associated with high BEDs could be considered an effective strategy in triggering the abscopal effect, thus shedding light on the promising outcomes revealed in clinical practice.

## Introduction

About 50–60% of solid tumors are treated with radiotherapy (RT) alone or in combination with surgery and/or other therapies such as chemotherapy, immunotherapy, hyperthermia to increase overall survival compared to single-modality therapy. Different RT treatments adopted in clinical practice aim to treat the disease sparing normal tissue from excessive toxicity. Accordingly, toxicity surrounding normal tissues along with several other factors, such as poor blood flow and hypoxia are commonly known to limit the success of traditional RT of bulky or deep-seated tumors.

Intra-Operative RT (IORT), or the Stereotactic Ablative RT (SABR), using high doses in a single or few fractions, named oligofractions, are becoming an attractive therapeutic option to improve cancer patient outcome [1,2]. In fact, technological advancements in terms of dose delivery and accurate setup of linear accelerators supplied with imaging devices allow more conformal dose delivery even at high dose rates. This has further encouraged radiation oncologists to adopt schedules of severe treatments with doses per fraction higher than 10 Gy and up to 20–30 Gy. From a radiobiological perspective, models and dose constrains adopted for conventional fractionation are currently under debate in terms of appropriateness and robustness, consequently stimulating researchers toward conducting confirmatory pre-clinical and clinical studies.

Importantly, studies from several laboratories are suggesting that RT with high dose per fraction (>8-10Gy) might trigger additional indirect biological effects besides the direct RT tumor cell killing. These indirect biological effects, initially described as abscopal and/or bystander effects [3, 4, 5] are now known to involve anti-tumor immunity, and/or vascular damage and/or other immunogenic forms of tumor cell death associated with the activation of signals leading to apoptosis, necrosis or necroptosis [6, 7]. Moreover, based on the linear quadratic (LQ) model, locoregional and distant tumor control by SABR or stereotactic radiosurgery [8] is underestimated when compared to clinical results. Interestingly, this discrepancy may likely be due to failing to take into account the significant contribution of the indirect biological effects [9, 10, 11].

In recent years, the abscopal effect (AE) has been sporadically observed in clinical cases (reviewed in [12, 13,14]) as well as reported in *in vitro* and preclinical studies [4, 5]. We believe that with current modern technologies, which allow the delivery of higher dose per fraction to tumors (such as in SABR treatment), the historical radiobiological models used for data description and outcome predictions could become obsolete or in any case limited, not including the activation of the tumor microenvironment. This hypothesis has not been fully explored and is intriguing for the potential outcome of modern RT treatments. Indeed, AE could be advantageous in high-risk cancer patients where therapeutic failure is mainly due to distant disease progression and not to local recurrence. Accordingly, results from our earlier studies have demonstrated that high RT dose (20 Gy) tumor delivery is required to trigger AE in preclinical mouse models [15]. Of note, our studies focused on the role of the biologically effective doses (BED) that is a parameter related to the expected survival used to compare and estimate the efficacy of different RT schedules [16]. Since 1989, BED has been introduced to compare the effects of different fractionations in radiotherapy in terms of cell killing [17]. By definition, BED is higher when the dose per fraction increases with the same total dose i.e. when schedules are more biologically effective.

Indeed, our experimental results [15] suggestively indicated that scheduled treatment and the total radiation dose may be important determinants of AE.

In the present study, with the aim of identifying the existence of a possible BED cut-off, capable of triggering AE, we reviewed the most relevant studies involving animal models and reanalyzed the reported data from a radiobiological point of view. In this regard, we evaluated whether the schedule, the delivery modality (RT regimen, irradiation source, etc.), the BED and the total dose of RT may be crucial determining factors of AE in preclinical models. The main purpose was to evaluate whether the role of delivered dose in promoting the AE had been considered in previous studies and what observations could arise from a critical reanalysis of published data. It is well accepted, in fact, that apart from direct DNA damage, additional molecular and cellular pathways are important for the clinical response to local RT, including an active cross-talk between the tumor microenvironment and the immune system. Of relevance, data generated from preclinical models might provide important insights into the potential mechanisms of the radiation-mediated AE, which can be further exploited for improving clinical treatment.

## Materials and methods

### Search strategy and selection criteria

The main aim of our search was to recover and reanalyze published data on distant tumor response after treatment with RT alone on primary tumor to evaluate AE occurrence. To investigate the role of the delivered dose in cancer treatment and AE occurrence based on *in vivo* murine preclinical studies, we performed a literature search on PubMed using the broad search terms: “abscopal” AND “animal model OR mouse OR preclinical” from 1954 up to 2015. The date of the search was 9 February 2015.

The PRISMA methodology was used for selecting studies based on the following criteria. Titles and abstracts were reviewed independently by two authors for the inclusion, and in case of controversial judgment, the paper was evaluated by a third author. Full articles were retrieved when the abstract was considered relevant and only papers published in English were considered. The bibliographies of retrieved papers and reviews were also sought to identify other relevant articles to be included. Papers were considered eligible when reporting data, graphs/figures on tumor volume growth over time of non-irradiated tumors (NIR) and control groups (no treatment) after RT alone. In other words, only data related to indirectly RT-treated or untreated (control group) animals were considered for data analysis, excluding groups directly irradiated or undergoing combined chemotherapeutic treatments or receiving injection/administration of immuno-modulators or other immuno-stimulatory substances. In any case, the information of the combined approach used has been reported for completeness in Tables 1 and 2.

**Table 1:** Nine preclinical studies out of 14 reported data on observed abscopal effect in the non-irradiated controlateral tumor.

Ref.	Mouse strain	Cancer type (cell line)	Biological endpoint	Immune therapy	Mediator of the abscopal effect (proposed)	Irradiated site	RT dose schedule (dose/fr X number of fractions)	BED (Gy10)	Abscopal effect of RT alone on NIR	Notes
**[20]**	C57BL/6	Lung (LLC-LM)	Tumor growth	none	p53	hind leg	10 Gy x 5	100	1	
							2 Gy x 12	28,8	1	
	C57BL/6	Fibrosarcoma (T241)	Tumor growth	none	p53	hind leg	10 Gy x 5	100	1	
							2 Gy x 12	28,8	1	
**[21]**	BALB/c	Breast (67NR)	Growth delay	Flt3-L	DCs, T cells	flank	2 Gy	2,4	0	the abscopal effect was shown to be tumor specific
**[22]**	BALB/c	Breast (TS/A)	Growth inhibition of NIR	anti-CTLA-4-mAb (9H10)	CD8+ and CD4+ T cells, INF-gamma	flank	20 Gy × 1	60	0	more effective at 8 Gy in combination with mAbs
							8 Gy × 3	43,2	0	
							6 Gy × 5	48	0	
	C57BL/6	Colon (MCA38)	Growth inhibition of NIR	anti-CTLA-4-mAb (9H10)	CD8+ and CD4+ T cells, INF-gamma	flank	20 Gy × 1	60	0	
							8 Gy × 3	43,2	0	
							6 Gy × 5	48	0	
**[18]**	NCr nu/nu	Pancreas (BxPC-3)	Tumor volume		cytokines or other innate immune mechanisms	flank	2 Gy x 10	24	0	xenograft; the other mice groups were treated with RT+capecitabine+colecoxib
**[23]**	C3H/He	squamous cell carcinoma (SCCVII)	Growth inhibition of NIR	i.t. DCs	DC, gp96	femur	10 Gy x 3	60	0	
**[24]**	BALB/c	Colon (Colon26)	Growth inhibition of NIR	ECI301	CD8+ and CD4+ T cells, NK1.1 cells, INF-gamma	6 Gy	9,6	0	6 Gy	the effect is tumor type independent; only at high dose of ECl301
	BALB/c	Sarcoma(MethA)	Growth inhibition of NIR	ECI301	CD8+ and CD4+ T cells, NK1.1 cells, INF-gamma	6 Gy	9,6	0	6 Gy	
	C57BL/6	Lung(LCC)	Growth inhibition of NIR	ECI301	CD8+ and CD4+ T cells, NK1.1 cells, INF-gamma	6 Gy	9,6	0	6 Gy	
**[15]**	CD1 nu/nu	Colon (HCT116 p53 wt; HCT116 p53-null)	Tumor volume	none	p53, RT dose	flank	10 Gy	20	0	
							20 Gy	60	1	
	CD1 nu/nu	Lung (A549^a^)	Tumor volume	none	p53, RT dose	flank	10 Gy	20	0	
							20 Gy	60	1	
**[25]**	BALB/c	Breast (TUBO)	Distant tumor growth inhibition	anti-PD-L1 (B7-H1)	CD8+ T cells	flank	12 Gy	26,4	0	reported only for TUBO
							20 Gy	60	0	
	C57BL /6	Colon (MC38)	Distant tumor growth inhibition	anti-PD-L1 (B7-H1)	CD8+ T cells	flank	12 Gy	26,4	0	
							20 Gy	60	0	
**[26]**	C3H/HeN	Breast (FM3A)	Distant tumor growth inhibition	ECI301	HMGB1	flank	6 Gy	9,6	0	

Relevant information and data are indicated, together with the calculated BED and the abscopal effect (AE) due to radiotherapy (RT) alone on the non-irradiated (NIR) tumor, according to the reported data on published figures and graphs. Immune therapy and the proposed mediator of AE are also included for completing information. i.t. = intra tumoral (injections)

^a^ unpublished data.

**Table 2:** Five preclinical studies reporting abscopal effect in murine model had different biological endpoints.

Reference	Mouse strain	Cancer type	Observed abscopal effect	Immune therapy	Proposed mediator	Irradiated site	RT dose	BED	P_AE_,_RT_	Notes
**[27]**	C57BL/6	Lung (3LL, D122)	lung metastasis regression	Flt3-L	DC	foot	60 Gy x 1	420	1,00	
**[28]**	C57BL/6	Melanoma (D5); Fibrosarcoma (MCA205)	inhibition of lung metastasis	i.t. DCs	DC	flank	8.5 Gy x 5	6,375	0,08	after adoptive transfer of splenocytes
**[29]**	BALB/c	Breast (4T1)	inhibition of lung metastasis	anti-CTLA-4-mAb (9H10)	CD8+ T cells	flank	12 Gy x 1	26,4	0,18	
							12 Gy x 2	52,8	0,40	
**[30]**	C57BL/6	Breast (4T1), Melanoma (B16)	Elimination of lung/inguinal lymph nodes metastases	none	CD8+ T cells	back	20 Gy x 1	60	0,48	With CD8+ cell depletion, the tumors become radio-resistant; effect on breast is less pronounced
**[31]**	BALB/c	Colon (Colon26)	decreased number of hepatic metastases	IL-2	CD4+ T cells	flank	2 Gy x 10	24	0,16	

Another five preclinical studies reporting abscopal effect (AE) observed in murine model and having different biological endpoints than distant tumor growth inhibition have been reviewed in this work, and the probability of observing the AE after a treatment of radiotherapy (RT) alone has been calculated (P_AE_,_RT_ column). i.t. = intra tumoral (injections)

Studies reporting on AE with different biological endpoints (i.e. decreasing number of distant metastasis) were also included for completeness of information but handled separately and reported in a different Table (Table 2).

Moreover, studies involving solid tumors were included, but excluded those using irradiated bone marrow cell transplantation, also studies using transgenic animal or strategy were not considered in this work. Review papers on the topic were also considered for identifying additional references.

In particular, data regarding the strain of animal models used in the experimental setting, the tumor type, the biological endpoint and the RT dose delivered were registered and discussed. We further reported and discussed the model system used in the experimental setting, the cancer type(s), and the referred mediator of the AE. Moreover, as described in the following sections, we calculated the BED and evaluated the AE of RT alone on NIR tumors comparing the tumor growth with the control.

### Tumor growth/control data extraction

Data on tumor growth delay or reduction of both control and NIR tumors were extracted directly from figures and graphs reported in the publications by using a home-made software. This software recovers original values (e.g. volumes/masses and time after irradiation for each graph) from published images by converting point distances into values of volume and time taking into consideration the unit scales on the axes. This tool permits the average tumor volumes and related uncertainties versus time to be evaluated. In particular, our program digitizes data, such as other free software available online that has been used for tool validation (such as: http://life.bio.sunysb.edu/morph/windig.html).

The y-scale was already in mm^3^ in most of the figures; in others the mass was converted from mg to mm^3^ assuming a density of 1 μg/mm^3^. Only in one case [18] the percentage value was converted into tumor volume (expressed in mm^3^) multiplying the extrapolated values by the median initial volume (i.e. using 37.5 mm^3^/100).

In all cases, the statistical significance of NIR volumes relative to controls was established on the basis of a statement/indication in the paper.

A score of 1 (indicating the occurrence of AE) was assigned when the NIR tumor volumes were statistically significant different from control, otherwise a score of 0 was given (see the column “Abscopal effect of RT alone on NIR” in Table 1).

### Data analysis

Data extracted from published works were further analyzed in order to calculate the BED according to the following formula:

$$\text{BED}=\text{D}\left(1+\frac{\text{d}}{\alpha /\beta }\right)$$

where D is the total dose; d is the dose per fraction; *α* and *β* are constant representing lethal and sub-lethal damage, respectively. The *α* parameter represents the initial slope of the dose response curve and residual not reparable radiation damage, while *β* represents the terminal slope and it is related to reparable events. In more detail, the *α* / *β* ratio allows the dose fractionation i.e. converting the total dose and the dose/fraction in a single number i.e. BED, to be taken into consideration.

For tumors considered in this paper, the *α* / *β* ratio has been assumed equal to 10 Gy, as usually adopted in clinical practice and largely reported in literature [16].

RT schedules were duplicated for each tumor cell line investigated in the selected studies, in order to weight the number of individual experiments conducted.

The probability of AE versus BED was obtained using the logistic regression model considering p< 0.05 as cut-off for the model.

The adopted function was:

$$\text{p}=\frac{1}{1+\text{exp}(\text{-}\;(ß0+ß1*\kappa *\text{BED})}$$

In this paper the parameter *κ* is assumed equal to 1, as reported for p53-wt cells [19].

Moreover, the BED_50_ is the dose for which the probability of observing the AE is 50%, assuming *κ* = 1.

The above equation could be generalized to include other models which assume *κ* < 1 [19]. Unfortunately, experimental data to adopt a κ value different from unity are still not available based on our pooled dataset. The use of *κ* < 1 decreases the BED_50_ (obtained with *κ* = 1) to a value *κ* * BED_50_i.e. translates the point of inflection of dose-response curve obtained with *κ* = 1.

The log-likelihood was calculated together with Chi-square. For each variable the coefficient and standard error were calculated. The fitted model and 66% confidence interval (CI) were plotted together with the experimental data.

To validate the model the bootstrap approach was used using 1000 resampling (b = 1000). Optimism of model and the bias-corrected Area Under the Curve (AUC) were calculated. The calibration curve was obtained using re-sampling and the mean absolute error and the mean squared error was derived. P-values lower than 0.05 were considered statistically significant.

## Results

**Fig 1:**
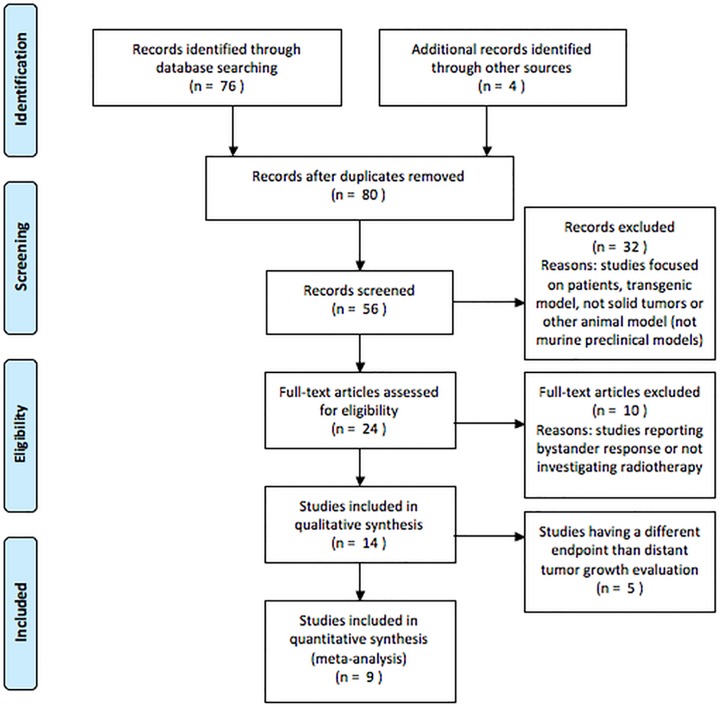
Study flow chart. Flow diagram of the study inclusion concerning papers published between 1954 and 2015.

A literature search on PubMed containing the terms “abscopal” AND “animal model OR mouse OR preclinical” yielded 76 publications (9 February 2015). Four additional studies were recovered by searching the bibliographies of retrieved papers and were included in the analysis. The selection criteria and exclusion reasons following the PRISMA statement are summarized in Fig 1 and PRISMA checklist is reported in **S1 File**.

According to our queries, a total of 14 preclinical studies reporting on AE were retrieved, the majority focused on combined RT and immune-therapy treatment. Nine out of 14 preclinical studies reported RT induced effect on directly IR and NIR tumor growth reduction/delay to assess AE. These studies met the inclusion criteria having recoverable data on contralateral tumor growth in the group of animals treated with RT alone and were considered eligible for data re-elaboration and BED calculation. Relevant information and data are summarized in Table 1, exploring also a putative involvement of the immune response and other potential molecular players, as evidenced in the original study.

The remaining 5 studies reported on occurrence of AE in preclinical models, but lacked in recoverable data. These studies, based on different biological endpoint, were included for completing information and as discussion points and are indicated separately in Table 2. Moreover, data from these studies were used to calculate the theoretical probability of observing the AE after a treatment of RT alone. The specific graph/figure of selected literature, used as data source, and additional information as experimental design and protocol are indicated in Table 3.

**Table 3:** Additional information regarding experimental design and radiation source reported in the reviewed studies.

Reference	Data source	Animal age (weeks)	Tumor size at the beginning of treatment (mm^3^)	RT after inoculation (days)	tumor growth monitored (days)	Radiation source
**[20]**	Fig 1	4–6	360±88	10	20	Gammacell Cesium 137
**[21]**	Fig 2	6–8	170–255	20	>50	^60^Co
**[22]**	Fig 2 (panel A)	6–8	32 (TSA) 50 (MCA38)	12	35	^60^Co
**[18]**	Fig 4 (panel B)	NR	35–40	28 and 52	50	^60^Co teletherapy X-ray unit
**[23]**	Fig 6	6–10	NR	6	30	NR
**[24]**	Fig 2 (panel A)	7	10 mm diameter	18	24	NR
**[15]**	Fig 3	nearly 6 (40 days)	100–200	21–28	56	Liac ® 12MeV for IORT
**[25]**	Fig 2 (panel D)	6–8	NR	14 (TUBO) 8 (MC38)	35	NR
**[26]**	Fig 6 (panel A and B -FM3A mammary)	7	10 mm diameter	15	10	NR
**[27]**	NA	6–8	≤100 mg / ≤6 mm in diameter	21	60	X-ray generator
**[28]**	NA	8	25 mm^2^	7-11/6-10	16	X-ray unit
**[29]**	NA	6–8	5 mm in diameter (~65 mm^3^)	13	35	^60^Co
**[30]**	NA	6–16	NR	7	21(40)	X-ray generator
**[31]**	NA	NR	1000 (about 1 cm in diameter)	21	20	X-ray generator

The specific graph/figure used as data source to evaluate the abscopal effect in non-irradiated tumors as reported in Table 1, as well as additional information regarding experimental designs and radiation source used in all the investigated studies (Tables 1 and 2) are reported. NA = not available; NR = not reported.

In selected studies, RT treatment schedules were duplicated for each tumor cell line investigated, in order to weight the number of individual experiments conducted. Through using this approach, 25 trials were identified and analyzed for preclinical studies having adopted secondary tumor growth reduction (Table 1), and 6 trials for the other group as endpoints (Table 2).

The AE occurrence on NIR tumors upon RT treatment was scored as 1, whereas a score of 0 was appointed when AE were not reported. Scores were then plotted against BED according to the investigated schedules (Table 1). Based on Table 1, the coefficients of logistic regression model were: ß0 = -3.4918 and ß1 = 0.0494±0.0244, with p-value of model of 0.015, and Chi-square coefficient of 5.93.

AUC was 0.80, and it was 0.79 after the validation procedure while a mean squared error = 0.104 was obtained by the calibration phase.

**Fig 2:**
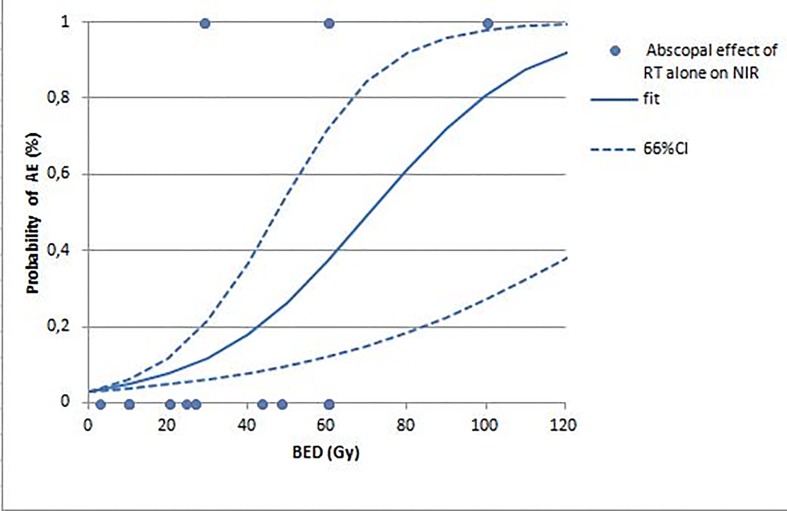
Relationship between BED and abscopal effect. The probability of observing the abscopal effect (AE) of radiotherapy (RT) alone out of the irradiated tumor target is the function of the biologically effective dose (BED). NIR = not-irradiated; CI = confidence interval.

Experimental data, plotted in the fitted curve (Fig 2), allow to identifying the percentage of probability revealing AE in correlation with the BED (Table 2).

Our results support that the rate of occurrence of AE in preclinical models is directly correlated with BED. In particular, at BED of 60 Gy, i.e. BED_50_, the probability of observing the AE is more than 50%. In greater detail, in considering the trials reported in Table 1, and assuming the logistic equation indicated in the methods section, the calculated BED ranges from 9.6 to 100 Gy, suggesting a probability of AE occurrence that ranges from 6% to 84%. Instead, according to the trials reported in Table 2, the calculated BED ranges from 6.4 to 420 Gy, indicating the probability of AE occurrence that ranges from 8% to 100%.

In general, in the selected studies reported in Table 1 and Table 2, the animal models used were immunocompetent mice, such as the BALB/c and C57BL/6 strains (10/14 studies). Two studies [15 and 18] reported AE in immuno-deficient strains and have been specifically indicated in Fig 2 using different dot symbols (closed squares). Considering the different experimental models adopted in selected studies, the analyses reveal that occurrence of RT-induced AE was described with different tumor cell lines of different histotypes: breast (6/14), colon (5/14), lung (4/14), fibrosarcoma (3/14), melanoma (2/14), pancreas (1/14) and head and neck cancer (1/14). Moreover, in the analyzed experimental models, most of the studies started with tumor cell implantation in 6–8 week-old mice, whereas in some other cases the age of animals was indicated within a rather broad range (i.e. 6–16 weeks, [30]) or not reported at all [18].

The analyses of the scheduled time selected to begin RT treatments in each study revealed that differences depended on the experimental setting, as well as the type of tumor under investigation, the tumor growth rate, or the excessive tumor burden. However, the scheduled time chosen could influence, to a different extent, the growth and the volume of the primary tumor treated, as well as the untreated secondary tumors, the magnitude of the involvement of the tumor microenvironment and/or other molecular/cellular players. Of relevance, the tumor volumes at the beginning of RT treatment are not always reported in these studies (see Table 3).

The overall treatment time and/or interval between fractions varied across the published schedules from 0 day (for single fraction, e.g. ref. [15, 21, 24–26, 27, 30]) to 10 days for longer schedules, i.e. ref. [18]. Being the overall treatment time less than 2 weeks the time factor was considered trivial in this paper.

Analyzing the dose rates adopted in RT treatments, 35% (5 out of 14) of collected studies scheduled ≥ 10 Gy, whereas a dose of ≥ 20 Gy was delivered in 43% (6 out of 14) of collected studies. Furthermore, the high dose was generally delivered in a single fraction, except for three cases where delivery was hypofractionated. The radiation source was reported in 43% (6 out of 14) of studies without any detailed information regarding the irradiation modalities. Only one study reported dose verification by using the in vivo dosimetry [15]. The use of on-line/off-line in vivo dosimetry tool allows further improving the dose delivery accuracy and further permit to control/appreciate the measured and expected dose.

## Discussion

The present study aimed to review all the literature describing AE through in vivo approaches focusing on exploring the dose-effect relationship. Indeed, we noted by graphs/figures that AE was appreciable also in NIR lesions of an animal group locally treated with RT alone, although most of the reviewed studies focused on the efficacy of combined regimes (i.e. RT and immuno-therapy/chemotherapy). Moreover, in most cases, NIR tumors after RT alone had a better tumor control over time than those treated with immuno-therapy alone.

The analyses of the experimental strategies adopted in the collected studies revealed differences in high dose radiation regimens and in subcutaneous implantation of tumor cell lines in syngeneic mice [9, 32]. Thus, we queried whether the dosage of the RT alone could play a critical role in inducing molecular/cellular effectors or altering the tumor microenvironment, hence producing an AE on NIR tumors. Then, according to our recent experimental findings [15], a threshold dose (BED) was investigated pooling retrieved data and comparing the resulted dose or BED with those of SABR approach reported to have a more effective clinical outcome than expected on conventional schedules.

In accordance with earlier studies [20], our results suggested that AE triggering is dose-dependent and more marked in hypofractionated radiation doses compared to standard RT dose schedules (2Gyx12). So, we investigated the potential relationship between the predicted AE probability and the BED, based on the LQ model of cell survival, hypothesizing a BED value capable of activating different cellular mechanisms able to trigger the AE.

Our experimental findings [15] are in line with those obtained in this paper based on the logistic regression model (Fig 2), which suggests greater probability of AE occurrence when BED increases, thus indicating the negative results of studies using RT schedules at low BED values. The model suggests that more attention should be paid to designing of RT schedules when AE is studied. A possible bias may be that when combined treatments are investigated, researchers are forced to set the effect of RT alone in order to avoid the saturation of the phenomenon, thus selecting doses or schedules at sub-optimal value. In this way, the combined agents under investigation could exploit their effects to a maximum.

It is noteworthy to highlight that from a radiotherapy and radiobiological perspective, all the schedules reported in the published studies on AE in preclinical models could be considered to be similar to fractionation adopted in the clinical practice for SABR.

Although the use of RT alone could be considered a limitation of the current study we would highlight that RT is a physical agent standardized worldwide from the point of view of dose measurement, allowing results from different centers to be compared using various dosages. Moreover, RT alone is currently applied in a large cohort of cancer patients, up to 50%.

Some issues need to be considered as potential confounding factors in the reviewed studies. Strains with different genetic backgrounds, radiosensitivity and immune-competence were used in these studies, all factors potentially influencing the response to ionizing radiation treatments [33, 34, 35, 36 37, 38]. Indeed, BALB/c mice are more radiosensitive and more susceptible than C57BL/6, and other tested inbred mice that develop mammary tumors upon ionizing radiation [39].

Burnette et al. [32] highlighted that in vivo studies rarely discuss other crucial aspect in the experimental setting, namely the required induction of an immunosuppressive tumor microenvironment to allow some cancer lines to establish lesions upon injection in syngeneic animals. Consequently, an immunosuppressive tumor microenvironment generates a progressive and fast tumor growth that generally causes a rapid death of the host within 3–6 weeks. Obviously in all the studies investigated the tumor injection site and the selection of mice strains were based on the possibility of establishing and following tumor growth, as an essential prerequisite.

**Fig 3:**
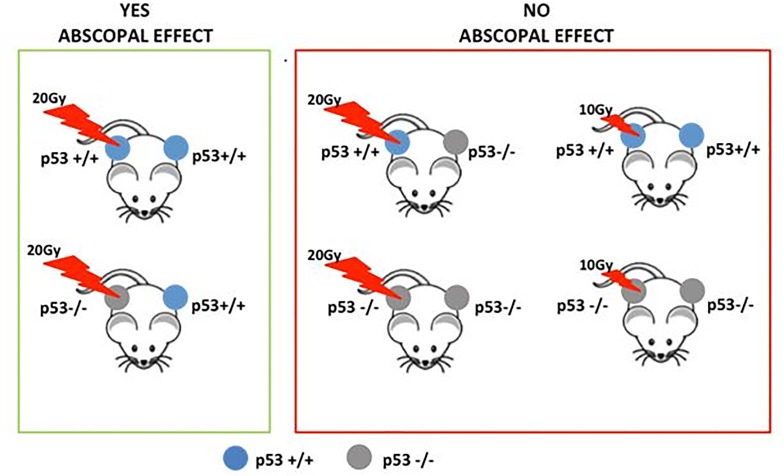
Abscopal effect and molecular player. Schematic representation of our results previously obtained on abscopal effect and p53 status in a preclinical model.

Different mechanisms have been proposed in literature to elucidate the occurrence of the AE, and the more accredited include immune reaction and vascular endothelium involvement [4, 5], which could also explain the higher efficacy of SABR treatment. A few other studies have suggested the relevance of dose-rate dependency or the occurrence of functional proteins, such as the well-known TP53, the “guardian of the genome”. Accordingly, in 2003 Camphausen and colleagues [20] demonstrated for the first time that wild type (wt) p53 is a key mediator of RT induced antitumor AE and suggested that it could not be tumor-specific. Their study also indicated a radiation-dose dependency for the occurrence of this effect. In fact they reported a reduction in distant tumor (lung carcinoma or fibrosarcoma implanted in the dorsal midline) after high dose RT delivered to the leg of immunocompetent mice, while an AE antitumor response was not observed in p53 knockout mice or in mice with p53 pharmacologically inhibited [20]. Our research group obtained consistent results in observing the effect in NIR tumors according the p53 status and delivered dose [15], as schematically summarized in Fig 3.

The effect of mut-p53 is also associated to the parameter κ different from unity [19] suggesting a change of BED_50_ value in our model. Of note, we highlighted that the occurrence of wt p53 in NIR tumors is required to trigger the AE, whereas IR tumors can directly release signals independently from the p53 status [15]. Another aspect to be highlighted is that we and another study [15 and 18] have reported AE in nude strains (athymic mice). We considered the results reported in these two studies of particular interest, since nude mice are partially immune-deficient. In fact, their vestigial thymus is incapable of producing mature T-cells, however they have T-cell precursors in their bone marrow. They also have impaired T-cell function as demonstrated by their failure to reject allogeneic and xenogeneic cells and tumor grafts. Of note, T-cell precursors in nude mice do not have a defect, and some functional mature T cells can be found especially in adult animals. Moreover, the population of mature CD8+ T-cells in nude mice is cytolytically active, their response to T-independent antigens is normal, even having an increased macrophage and natural killer response than those from BALB/c mice [40, 41]. Thus, AE results obtained in nude strains seem to suggest that other immune mechanisms may exhibit a role in AE. Undoubtedly, all these studies indicate that RT can elicit complex responses on tissues, and these responses may have systemic effects, which also depend of immune stimulation and the tumor microenvironment composition.

Of note, in the retrieved studies we recognized that the best outcomes in terms of local tumor control or rejection have been reported by combining RT with immuno-therapy, suggesting that local RT can induce a systemic antitumor immunity, such as an effective “in situ vaccination” [42], the RT being a pre-requisite to observe the AE.

In this context for instance, it should be considered that for the study [21] the efficacy of Fms-related tyrosine kinase 3 ligand (Flt3L)-induced antitumor immune response is known to be limited by tumor size [43]. Flt3L in combination with radiation resulted in the induction of tumor-specific T-cell immunity and the control of tumor growth outside of the irradiated field in two of the reviewed studies [17, 23]. In any case, in the cited study [21] the authors demonstrated that T cells are required for the AE obtained with the combination of local RT and Flt-3L treatment and suggested that tumor death might in part be associated with the release of cytokines and other inflammatory stimuli, which can promote the appropriate signals for dendritic cell activation. Another study [22] observed that only fractionated RT induced an AE in a secondary tumor when combined with anticytotoxic T-lymphocyte antigen-4 (CTLA-4) antibody immunotherapy. This study supports that the radiation regimen, site of RT and tumor characteristics may all contribute to the synergistic effects of RT and anti-CTLA-4. This synergy is currently under investigation in prospective clinical trials, especially to evaluate abscopal responses following RT in patients with metastatic melanoma treated with ipilimumab (therapy with CTLA-4 blockade) [44,45].

The dose-response effect based on the use of combined chemotherapy or immunotherapy with RT might require different investigations using the same drug/agent to be grouped and analyzed, as recently reported in a distinct paper [46]. Unfortunately, at the time of our search these data were not available to be analyzed with our approach.

Regarding the tumor type, studies included in our review focused on AE observed in different tumor types, namely lung, fibrosarcoma, breast, pancreas, melanoma, head and neck and colon cancer. For all of these tumors AE has been reported in some clinical cases, although preclinical studies evidence more or less pronounced responses. For example in the study of Lee and colleagues with CD8+ cell depletion, the melanoma tumors become more radio-resistant, while on breast this effect is less pronounced [30]. In their study antitumor immunity was also reported to be achieved by irradiation alone.

It is important to emphasize that AE could be of particular relevance for the microscopical spread of cancer that is not detected by the modern imaging devices due to their resolution limits, whereby 1 mm^3^ (minimum voxel size of clinical images) contains about 24.000 cells [47]. Accordingly, the RT at high doses might be more effective than conventional RT, since it can induce an effective systemic control of distant tumor micro-lesions presumably by increasing apoptotic response in cancerous cells. In this scenario the proposed dose-response relationship derived from preclinical studies suggests that the potential improved clinical outcome could rely on the AE and not only on the cell killing effect modeled using the LQ model. Obviously this is a simplified model in comparison to the complexity of cellular and molecular players driving the AE, but it focuses on the fact that dose is a trigger for the occurrence of AE, thus suggesting how to identify appropriate RT treatment schedules by using BED in order to increase the probability of this effect. Furthermore our model provides a better possible explanation of the improved results of SABR treatment schedules.

Case studies on AE have been sporadically reported on different cancer types [12, 13, 14] usually treated at conventional fractionation. The recently renewed interest regarding the immunogenic effect of RT and its possibility to induce distant tumor regression with clear and important clinical benefits has fostered the research efforts on this topic, as demonstrated by the increment in publications over the last few years. In particular, the group of Demaria and Formenti are currently conducting an interesting clinical trial in order to produce an objective abscopal response in patients with metastatic solid tumors [48]. Of note, as recently highlighted for clinical case reports in lung, liver and bone, the calculation of BEDs indicated that the abscopal responses of non-irradiated lesions are observed at sub-ablative doses only when radiation regimens are delivered in combination with immunotherapy [49].

Several preclinical studies have focused on immune-mediated AE [21, 22, 24, 50], and although secreted soluble factors were previously found to be involved in this effect [51], the exact abscopal mechanisms remain to be elucidated [5, 52, 53]. Candidate effectors have been proposed among modulates cytokines/chemokines, immune system involvement, tumor-infiltrating cells, but their relative importance in driving AE seems to overly depend on the experimental model adopted and/or exposure conditions [54, 55, 56, 57]. Therefore, the identification and the understanding of the molecular/cellular players and signaling pathways involved in AE could be crucial for improving the efficiency of SABR/RT in clinical practice [54, 58, 59, 60].

## Conclusions

In the present study, we intended to focus further attention on the most important preclinical studies conducted on mice models, since we suppose that the heterogeneity of study designs, biological endpoints under investigation, combinations of immunological treatment with RT, and other aspects could, when all presented, have confounding factors in the understanding of the putative molecular mechanisms triggering the AE. Nonetheless, the heterogeneity emerging from these studies, the doses used in most of the experimental settings are comparable with those adopted in clinical practice, allowing better interpretations of the results for translational purposes. Interestingly, we believe that a critical review of preclinical tumor models adopted to study RT response could help to better mirror clinical situations, design more focused and accurate preclinical research and ease translation of experimental study results into clinical trials.

## Supporting information

S1 File PRISMA checklist. (DOC)
